# Malnutrition and disability: unexplored opportunities for collaboration

**DOI:** 10.1179/2046905514Y.0000000156

**Published:** 2014-04

**Authors:** N Groce, E Challenger, R Berman-Bieler, A Farkas, N Yilmaz, W Schultink, D Clark, C Kaplan, M Kerac

**Affiliations:** 1 Leonard Cheshire Disability and Inclusive Development Centre, University College London, UK; 2 Disability Section; 3 Nutrition Section, UNICEF, New York; 4 Spoon Foundation, Portland, Oregon, USA

**Keywords:** Malnutrition, Disability, Nutrition, Child health, Human rights, Public health, Global health, Delivery of health care

## Abstract

There is increasing international interest in the links between malnutrition and disability: both are major global public health problems, both are key human rights concerns, and both are currently prominent within the global health agenda. In this review, interactions between the two fields are explored and it is argued that strengthening links would lead to important mutual benefits and synergies. At numerous points throughout the life-cycle, malnutrition can cause or contribute to an individual’s physical, sensory, intellectual or mental health disability. By working more closely together, these problems can be transformed into opportunities: nutrition services and programmes for children and adults can act as entry points to address and, in some cases, avoid or mitigate disability; disability programmes can improve nutrition for the children and adults they serve. For this to happen, however, political commitment and resources are needed, as are better data.

## INTRODUCTION

Until recently, the policy, programme management and professional ‘worlds’ of malnutrition and disability have been largely separate. There is, however, increasing international interest in the links between the two.^1^–^3^ Sharing many common features, there is hope that closer links and stronger working relationships between the two fields could lead to important synergies and mutual benefits:

1. both fields address global problems affecting large numbers of often vulnerable individuals, including children and adults: some one billion people worldwide are malnourished,^4^ and around one billion live with a disability;^5^
2. both are related to key human rights: the right to food, a key determinant of nutritional status, is articulated in the 1948 Universal Declaration of Human Rights (Article 25);^6^ the General Comment on the Right to Food specifically mentions the rights of people with disabilities to have physical access to adequate food;^7^ the right of all children to adequate nutrition is laid out in the Convention on the Rights of the Child which has been ratified by all but two countries (http://www.unicef.org.uk/UNICEFs-Work/UN-Convention/) to health-care, education and social participation are articulated in the 2006 UN Convention on the Rights of Persons with Disabilities (CRPD) which has now been ratified by 147 countries;^8^
3. both are currently prominent within the global health agenda: the first ever World Report on Disability was published jointly in 2011 by WHO and the World Bank;^5^ cost-effective interventions for tackling malnutrition have recently been highlighted in the 2013 Lancet Nutrition Series;^9^ and Scaling-up Nutrition (SUN), launched in 2010, is a major new movement tackling malnutrition by ‘uniting people — from governments, civil society, the United Nations, donors, businesses and researchers — in a collective effort to improve nutrition.’^10^

In this narrative review, these and other critical links between malnutrition and physical, sensory (i.e. deafness, blindness), intellectual and mental health disability are explored. By providing a conceptual framework and identifying key areas where they interact, it is hoped to stimulate future work and, once a larger evidence base begins to be generated, to encourage future systematic reviews to guide policy and practice.

## MALNUTRITION AND DISABILITY: A FRAMEWORK

**Figure 1:**
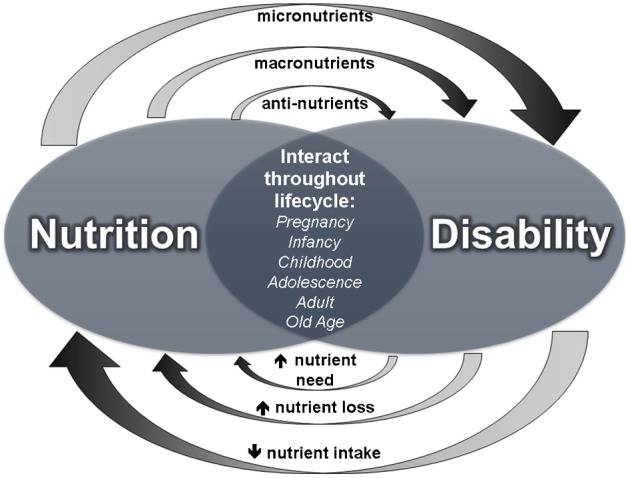
A conceptual framework of how malnutrition and disability relate and interact (adapted from Kerac *et al.*, 2014^1^)

The fields of malnutrition and disability are closely interrelated with a number of points of convergence. Countries with high levels of malnutrition and nutrient deficiency also often report higher rates of disability and developmental delay.^11^ There are several important areas of overlap and influence: malnutrition can cause or contribute to a variety of different disabilities; disabilities can cause or contribute to malnutrition. A conceptual framework showing the major pathways by which this occurs is shown in Figure 1.

Interactions occur throughout life — during pregnancy, infancy and childhood, adolescence, adulthood, and into old age. This paper will focus mainly on under-nutrition causing disability, and on disability causing or contributing to under-nutrition, although it is important also to recognise that overnutrition (obesity)-associated conditions such as stroke and diabetes are also increasingly important causes of disability, notably in older age-groups.^12^

## MALNUTRITION CAUSING AND CONTRIBUTING TO DISABILITY

This can occur throughout the life-cycle, the earliest effects being those of maternal malnutrition on the developing fetus. These early life opportunities to benefit future health and development are particularly important since not all adverse impacts of early malnutrition are reversible.

### Maternal malnutrition

Maternal malnutrition can affect the development of the fetus, cause intra-uterine growth delay and increase the risk of the infant developing impairments. Micronutrients often play specific roles in such occurrences. For example, low maternal folate is associated with an increased risk of neural tube defects, one of the clearest examples of a micronutrient-specific, often serious and yet largely preventable disability. One recent review concluded that ‘If folic acid food fortification achieved 100% population coverage, the number of neural tube defects in low-income countries could be approximately halved.’^13^ Vitamin D and calcium are other micronutrients implicated in disability. Deficiency of either during pregnancy is a risk factor for pre-term birth which is associated with numerous complications and adverse long-term sequelae including cerebral palsy and cognitive, visual and hearing impairments.^14^ Iodine deficiency is a major global cause of impaired cognitive development, the problem being most severe if the deficit occurs during early pregnancy.^15^ Iron deficiency is also common and can affect fetal brain structure and function, leading to cognitive and behavioural impairments.^16^ The timing of the deficit matters: animal models demonstrate that prenatal iron deprivation affects ‘activity, impulsivity and wariness whilst postnatal deprivation impairs emotional and cognitive development.’^17^ There is also good evidence of the effect of iron deficiency on human neuro-disability. One review notes the effect of treatment: ‘the short-term improvements seen in iron supplemented infants suggest that adverse effects can be prevented, reversed, or both.’^17^ However, the review also warns that the risk of iron-associated disability might not revert completely to a normal level after supplementation: ‘infants with iron deficiency anaemia are developmentally at risk in the short term and … continue to be so in the long term despite iron therapy.’^17^

A more general combination of maternal macro- and micronutrient malnutrition is associated with physical and neurological/cognitive disabilities. Sub-optimal pelvic growth in girls is one outcome of poor early nutrition^18^ and a risk factor for cephalo-pelvic disproportion, a cause of obstructed labour, and of fetal injury and birth asphyxia, both of which are major causes of cerebral palsy.^19^ This is a particular problem in resource-poor countries where, as well as higher rates of under-nutrition, there are also poor obstetric and neonatal services and hence a greater likelihood of perinatal difficulties resulting in more permanent problems. Evidence for malnutrition-related neurological and cognitive disabilities comes from studies such as those arising from the World War II ‘Dutch hunger’.^20^ Numerous studies have followed this cohort who were exposed to malnutrition *in utero*. Long-term effects included impaired cognitive performance, increased response to stress and an increased risk of schizophrenia.^21^ Physical effects of this early-life malnutrition are indirectly relevant to disability: coronary heart disease and diabetes are more common in the Dutch hunger survivors.^22^ These are well recognised risk factors for stroke which is a major cause of adult-onset disability.^23^ A similar but less well known study from an African setting followed a cohort exposed *in utero* to famine conditions during the 1967–1970 Nigerian civil war.^24^ As adults, they were at significantly increased risk of hypertension and impaired glucose tolerance than a control group born after the famine. Again, this has worrying implications for adult stroke and cardiovascular-related disability and highlights the need to tackle root causes as well as immediate consequences of disability.

Finally, the importance of maternal nutrition does not end with birth. Breast-milk is important for general development, and specific nutrients including vitamin B_12_ play a role in development of the nervous system. For example, maternal B_12_ deficiency (commonly caused by untreated pernicious anaemia or a strict vegan diet) with consequently low levels in breast-milk can lead to developmental delay and neurocognitive impairment. In one review of 18 low- and middle-income countries, children who were not breastfed during infancy were more likely to have positive findings at ages 2–4 years when screened for any disability using the UNICEF ten-question screen which enquires about speech, cognition, hearing, vision, motor/physical and seizure disorders.^26^

### Child nutrition

Infants and young children who are malnourished as defined by underweight (low weight-for-age) and stunting (low height-for-age) are also more likely to screen positive for disability.^26^ Macronutrient and micronutrient deficiencies are risk factors for physical, sensory and cognitive impairment.^1^,^27^ For example, regarding micronutrient-associated disability, each year between 250,000 and 500,000 children become blind as a result of vitamin A deficiency.^28^ Several of the B vitamins are associated with disabling conditions: vitamin B_1_ (thiamine) deficiency manifests as beri-beri, symptoms of which include a lower extremity polyneuropathy;^29^ vitamin B_3_ (niacin) deficiency manifests as pellagra whose neurological effects include confusion and agitation;^30^ vitamin B_6_ (pyridoxine) deficiency is a rare but well recognised cause of intractable epilepsy.^31^ Iodine deficiency affects the cognitive development of young children, with effects on very young children being the most severe and sometimes irreversible.^15^ Iron deficiency in children is associated with cognitive, learning and behavioural impairment.^32^

Childhood macronutrient malnutrition often manifests as underweight or wasting and also impairs immune system function and renders a child more susceptible to infection.^33^,^34^ Some infections, meningitis in particular, when treated incorrectly or late, as can often happen in resource-poor settings, are major causes of disability.^35^ Another manifestation of malnutrition is stunting, defined as height-for-age below two standard deviations of the median. It is caused by a range of nutrition-related determinants including macro- and micronutrients, and has a number of negative impacts on physical and cognitive development.^36^,^37^ Hearing loss is another possible malnutrition-linked disability. It has been found that infants with mild malnutrition are more likely to suffer hearing loss than infants who are not under-nourished, and the risk of hearing loss is increased in infants with severe-to-profound malnutrition.^38^

### Adult and later-life malnutrition

Obesity is rapidly emerging as a public health problem even in resource-poor countries.^39^ Early-life under-nutrition is an additional risk factor for obesity faced by societies in transition.^40^ With obesity comes the increased risk of metabolic disease and stroke, stroke being the third leading cause of disability-adjusted life years (DALYs) worldwide. Malnutrition and under-nutrition in older adults can also increase the likelihood of breaking bones, including hip fractures, which can lead to limited physical mobility; problems with physical mobility after illness or injury can leave older adults physically unable to obtain or prepare food for themselves; leading to changes in eating patterns which can lead to further disability in older adults.^23^

### Anti-nutrients

Anti-nutrients can affect individuals of any age. During times of food shortage, children and adults eat unfamiliar foods and do not know how to properly prepare them to make them safe for consumption and thus toxins which can cause neurological damage are not removed. Cyanide in bitter cassava is the best known example. This is normally removed by careful preparation: washing, drying, pounding and cooking. Failure to do this can cause permanent peripheral polyneuropathy which is well described in the literature.^41^,^42^ Spastic paraparesis is another disabling outcome of toxin ingestion: *lathyrism* is associated with the grass pea toxin beta-N-oxalyl-alpha, beta-diaminopropionic acid (beta-ODAP).^43^,^44^ *Konzo*, which develops rapidly and affects large numbers of people in epidemics, is also caused by poorly processed bitter cassava which contains cyanogenic glycosides and their metabolic by-products.^45^,^44^

## DISABILITY LEADING TO MALNUTRITION

With the proliferation of nutrition-disability associations described in the previous section, it is understandable that in the past many programmes focused on the prevention of disability through nutritional intervention. Whilst this will always remain significant, it is also important that future programmes recognise causality in the other direction: individuals who are born with or acquire a disability often face significant issues related to nutrition. Disabilities placing an individual at particularly high risk of nutritional deficiency include cerebral palsy, craniofacial anomalies (cleft lip and/or palate) and the many genetic syndromes such as Down syndrome and Pierre Robin sequence which are associated with, for example, oral–motor feeding and swallowing problems.

A high incidence/prevalence of malnutrition is often reported in children with disability,^46^,^47^ and this may result in poorer health and development, leading to a perpetuating cycle of sub-optimal nutrition, disability and worsening health status.^48^ In extreme cases, death can occur. A study in Malawi followed children treated for severe malnutrition and found that having an underlying disability (cerebral palsy being the commonest) was second only to HIV as a risk factor for mortality.^49^ Possible reasons for this include delayed presentation at a health or nutrition facility, sub-optimal care (i.e. not focused on specific needs of disabled children), more severe malnutrition on admission, and lack of follow-up after discharge from the nutritional treatment facility.

### Causal pathways – medical

In some newborns and children with disability, the impairment is a direct cause of malnutrition. There may be anatomic or oral motor/mechanical difficulties, leading to decreased nutrient intake. For example, a cleft palate affects sucking, chewing and feeding. Some 90% of children with cerebral palsy have difficulty feeding, which can result in malnutrition, poorer health status and sometimes even early death.^50^

Malabsorption of nutrients is also common in children with certain conditions, including cystic fibrosis. Unless carefully managed with specially adapted diets (including pancreatic enzyme supplementation in the case of cystic fibrosis), both macro- and micronutrient-related malnutrition can occur. This may lead to increased muscle wasting and loss of function, and further exacerbate the insufficient intake of energy and nutrients, now through mechanical causes.

Some children with disabilities may need additional nutrients to cope with the health problems associated with their disability. For example, a child with a physical disability may be prone to pressure sores caused by immobility or poor nursing which can become seriously infected. A high-quality diet is needed for prompt healing and to control infection. Poor families may struggle to meet these increased nutritional needs or lack ready access to the necessary dietary supplements.

Although the focus of concern for children with disability is generally around under-nutrition, becoming overweight is also a concern for some disabled children. For example, children with certain physical disabilities are less mobile and therefore at risk of becoming overweight.^38^ Other children with certain types of genetic impairments (e.g. Sotos syndrome) or intellectual or mental health disabilities may have eating disorders which place them at greater risk of becoming overweight. Overweight does not mean that all parts of the diet exceed normal needs: micronutrient deficiencies can occur in a high-energy but poor-quality diet and go unnoticed by parents and health-care workers, with serious long-term consequences for health and development.

### Causal pathways – educational

Parents, carers and service-providers may lack knowledge of how to feed their disabled child effectively or of how to teach the child to feed itself. This is especially important for children with conditions such as cerebral palsy who may need special seating or positioning to control muscle spasms or for children with Down’s syndrome who are at increased risk of choking and developing pneumonia. Difficult meal times can lead to increased stress levels for the caregiver and the child, which can result in insufficient food intake.^50^ Another concern related to improper feeding is the heightened risk of aspiration which can cause pneumonia and ultimately be life-threatening, particularly in children who are more generally vulnerable.

### Causal pathways – attitudinal, cultural and social

There is a common assumption that children with disabilities do not grow and thrive as a result of their impairment. Whilst some conditions are certainly associated with different growth ‘norms’,^51^ it is also important to acknowledge attitudinal, social and cultural factors. These may sometimes fully, sometimes partially explain sub-optimal nutrition. For example, in some cultures, mothers of newborns with a disability may be discouraged from breastfeeding, assured by family and midwives that the child will die anyway or will not lead a productive life. This becomes a self-fulfilling prophecy as these newborns will quickly starve. Stigma surrounding disability may result in children with disabilities being given less nutritious or smaller quantities of food, or intentionally not being fed at all, with families rationalising that limited resources should be devoted to children who have a greater chance of surviving and contributing to the household.^52^ This reasoning is not only incorrect but denies these children the right to grow and thrive.

Feeding practices are also of concern. Family members may treat a child with disability as an infant, continuing to give a liquid-only diet, believing that the child will not be able to take solid foods. This can lead to severe malnutrition and, in extreme cases, death.^48^

Parents and carers may also assume that a child with a disability cannot learn and develop in the same way as other children, and therefore such children may not be encouraged to feed themselves. Children with disabilities may therefore rely more on family members for food, placing severe time constraints and economic burdens on the caregivers and the household.^53^ The burden of feeding children with disabilities is also a gender issue as the responsibility of feeding such children falls most heavily on female members of the household who must balance the needs of the child with all other necessary chores. The result is that the child may receive less food because it is time-consuming to feed a child with low muscle tone and spasticity and no-one in the household has the time or energy to provide such feeding. This is an issue not only in the home. The UK-based international NGO Lumos recently reported that, owing to time and work constraints, caretakers of children with disabilities in Bulgarian institutions were unable to devote more than 2 minutes to each child during meal-times, despite the fact that these children needed help with feeding and drinking.^54^

Girls with disabilities may more often be underweight than boys with disability.^47^ In disadvantaged communities experiencing limited resources and food shortages, families following culturally determined gender preferences may choose to prioritise the nutritional needs of a disabled boy over that of a disabled girl. As noted earlier, this may have inter-generational effects: poor nutrition can lead to sub-optimal pelvic growth in girls with the increased risk of future children also having a disability. As more than half of all girls with disabilities will have children of their own,^55^ the potential inter-generational impact of such sub-optimal pelvic growth is considerable.

Health and social systems to improve the nutrition of children may be less accessible to those most in need, such as children with disabilities, and particularly those living in poverty or remote locations.^56^ Community-based health or nutrition services may be accessed less by children with disabilities owing to difficulties associated with reaching them (inaccessible or expensive transport, inaccessibility of buildings), or a lack of understanding by nutritionists and service-providers or by families and care-givers about appropriate interventions. In addition, many child nutrition campaigns are run through schools, and children with disabilities are less likely than non-disabled ones to attend school at all ages.^57^ This results in children with disabilities not only lagging behind their peers in educational attainment but also not benefiting from school-based nutrition initiatives. In extreme cases, some families or communities may place a lower status on a disabled child and prioritise nutrition and health services for their non-disabled siblings. Children with disabilities are also disproportionately represented in many institutions and orphanages, and these facilities are often overlooked in food programmes. An additional concern is the often poor quality of food in institutions. While of concern to all institutionalised children, children with disabilities may be at particular risk.

Finally, disability is often seen as a specialist subject and therefore not mainstreamed into education for practitioners in nutrition, health and child development. Pre- and in-service training of professionals in health-care, nutrition and development on the links between disability and nutrition would increase awareness of the specific nutrition requirements of children with disabilities, and expand more inclusive programmes and practice. As nutrition efforts are scaled up, the needs of children and adults with disabilities must be integrated to ensure that they are offered the same life-saving interventions as other children.

Nutrition and disability are intimately linked: malnutrition can directly cause or contribute to disability, and disability can lead to malnutrition. This has inter-generational and life-course implications. Though infants and children are especially vulnerable, older children and adults are also at risk, not least because early life malnutrition has long-term effects. Risk factors leading to malnutrition and disability are multi-faceted and encompass biological, physical, environmental and social factors.

Similar concerns surround the nutritional needs of adults with disabilities. When living in households with relatives, particularly where resources are scarce, the amount of nutritional food and water provided to them is often limited. Women are usually expected to assist with feeding and they have to balance this with many other demands on their time. Hundreds of thousands of disabled adults live in institutionalised settings in which access to adequate and nutritious food is often even more difficult and staff have limited time and energy to assist with feeding.

It is imperative that future nutrition policy and programming, maternal and child health, disability policy and broader public health initiatives recognise and plan for the malnutrition–disability link. By so doing, many current problems can be transformed into opportunities to benefit both areas of health-care. For this to happen, resources are needed and effective action planning is required. This would have important two-way benefits: disability programmes have great potential to serve as an entry point to nutrition services; similarly, nutrition programmes have the potential to act as entry points to disability services. Nutrition interventions for all children and adults will also benefit children and adults with disabilities. In order to ensure effective and inclusive nutrition, special attention should be paid by nutritionists, health-care and community service-providers to include children at high risk of becoming malnourished (such as those with existing disability or chronic disease) in existing nutrition programmes, as well as adapting or expanding community-based models of care and reaching out to institutions in which some children and adults with disabilities live.

Finally, children and adults with disabilities must also be included in general food security and treatment interventions to ensure they receive the best access to nutrition as a matter of equality and basic human rights. As children and adults with disability may often also require additional nutritional intervention specific to their impairment, issues of disability should be fully integrated into nutrition programmes, policies and services so that malnutrition and disability can be addressed jointly in daily life or during emergency food security crises.

## RECOMMENDATIONS

Whilst some international organisations, policy-makers and other members of the international community are beginning to recognise the importance of improved linking of nutrition and disability, much still needs to be done. As key steps forward, the following are suggested:

- The international community – governments, policy-makers, multi- and bi-lateral donors and practitioners – must ensure political and resource commitment to tackling nutrition and disability as related issues.
- To understand the links between nutrition and disability, better data are needed. This includes disaggregated data to enable comparison between the disabled and their non-disabled peers.
- International and national nutritional plans and policies must be explicit about disability-related links and interactions. One opportunity is during the roll-out of forthcoming new WHO guidelines on the management of severe acute malnutrition (SAM).^58^ These clearly, albeit briefly, recognise disability as an underlying cause of SAM which needs to be considered and addressed. As this international-level guidance is translated and adapted for national use, it is vital that the messages on disability remain strong. Ideally, national SAM guidelines should not only mention disability but also provide detailed disability-specific guidance.
- There is a need for improved access to nutrition services for pregnant and breastfeeding mothers, including disabled ones.
- Disability should be mainstreamed in all early-intervention nutrition, health and development efforts; for example, early screening for malnutrition should be adapted to ensure that it is more accessible to children with disability and their families than is currently the case.
- For some children with disabilities as well as for adults with disabilities, there is also a need for disability-specific services which target and address their needs and those of their families or caretakers, including professional special and community-based rehabilitation services where these are available.
- Development professionals as well as those training in health-care and nutrition should be educated in how to include children and adults with disabilities.

## DISCLAIMER STATEMENTS

**Contributors** NG, EC and MK provided initial draft and coordination of review. All co-authors were involved in drafting, refining and reviewing this paper throughout the writing process.

**Funding** Provided by UNICEF.

**Conflicts of interest** None.

**Ethics approval** None.
